# An Action Classification Method for Forklift Monitoring in Industry 4.0 Scenarios

**DOI:** 10.3390/s21155183

**Published:** 2021-07-30

**Authors:** Andrea Motroni, Alice Buffi, Paolo Nepa, Mario Pesi, Antonio Congi

**Affiliations:** 1 Department of Information Engineering, University of Pisa, Via G. Caruso, 56122 Pisa, Italy; paolo.nepa@unipi.it; 2 Department of Energy, Systems, Territory and Constructions Engineering, University of Pisa, 56122 Pisa, Italy; alice.buffi@unipi.it; 3 Institute of Electronics, Computer and Telecommunication Engineering (IEIIT), Italian National Research Council (CNR), 10129 Turin, Italy; 4 Sofidel SpA, 55016 Porcari, Italy; mario.pesi@sofidel.com (M.P.); antonio.congi@sofidel.com (A.C.)

**Keywords:** cyber-physical system, Industry 4.0, internet-of-reader, IREAD 4.0, radio frequency identification, RFID classification method, smart gate, smart forklift, smart warehouse

## Abstract

The I-READ 4.0 project is aimed at developing an integrated and autonomous Cyber-Physical System for automatic management of very large warehouses with a high-stock rotation index. Thanks to a network of Radio Frequency Identification (RFID) readers operating in the Ultra-High-Frequency (UHF) band, both fixed and mobile, it is possible to implement an efficient management of assets and forklifts operating in an indoor scenario. A key component to accomplish this goal is the UHF-RFID Smart Gate, which consists of a checkpoint infrastructure based on RFID technology to identify forklifts and their direction of transit. This paper presents the implementation of a UHF-RFID Smart Gate with a single reader antenna with asymmetrical deployment, thus allowing the correct action classification with reduced infrastructure complexity and cost. The action classification method exploits the signal phase backscattered by RFID tags placed on the forklifts. The performance and the method capabilities are demonstrated through an on-site demonstrator in a real warehouse.

## 1. Introduction

The term “Industry 4.0” was born in 2013 when the German government promoted the “High-Tech Strategy 2020 Action Plan” for a planned “4th industrial revolution” [1]. Since then, notable efforts have been carried out toward the implementation of Smart Factories [2] and Smart Warehouses [3]. The underlying concept concerns the integration of industrial technologies with information and communication technologies, which leads to the implementation of a Cyber-Physical-System (CPS) [4]. Each part of the system becomes able to autonomously exchange information, trigger actions and control each other [5]. In other words, a CPS allows the implementation of a digital and intelligent factory in order to promote manufacturing to become more digital, information-led, customized, and green [6]. Furthermore, several enabling technologies have been developed for the Industry 4.0 paradigm, e.g., Internet of Things (IoT) [7], Near-Field Communication (NFC) [8], Radio Frequency Identification (RFID) [9], Wireless Sensor Network (WSN) [10], and Block Chain (BC) [11], to name but a few.

The last few years have seen more widespread diffusion of solutions and systems put into practice for the fourth industrial revolution. The aim is to implement an interconnection between production facilities, storage systems, and factory machinery in such a way to allow a real-time interaction between workers, devices and items in the whole supply chain. Consequently, both factory and warehouse facilities may become *smart*.

In such a framework, the implementation of a smart warehouse concerns two different aspects. From one side, the possibility of a real-time inventory of items within the warehouse allows the definition of a proper company production-plan based on the market demand, by avoiding excesses of production and warehouse congestion. On the other hand, the development of a location-based system makes sure of not only the awareness of the item presence but also of its position within the warehouse, together with the position of the vehicles employed for procurement operations. It follows the development of lots of additional functionalities such as the optimization of item placement and of the vehicle paths during the loading/unloading operations with a consequent improvement of operator work-quality and safety.

The I-READ 4.0 project, funded by Regione Toscana, Italy, fits into this context. In particular, it concerns the implementation of an integrated and autonomous CPS for the automatic management of very large warehouses. The system consists of a network of RFID readers in the Ultra-High-Frequency (UHF) operating band, which are able to automatically collect data from the warehouse pallets equipped with UHF-RFID tags and stored within the tissue-paper warehouse of the Sofidel Italian Company in Porcari, Lucca. Firstly presented in [12], the I-READ 4.0 system consists of two main technological elements: UHF-RFID Smart Gate and UHF-RFID Smart Forklift. The Smart Gates use fixed readers able to detect forklifts/pallets entering or exiting from areas of interest. The Smart Forklifts are equipped with UHF-RFID readers able to auto-localize themselves by exploiting data from UHF-RFID reference tags in the scenario and then localize the tagged pallets in the indoor warehouse. The system is low-cost, reconfigurable, flexible and scalable regardless of several factors, e.g. warehouse sizes, good typology and spatial resolution required for item localization.

In this paper, the main idea of the I-READ 4.0 system is a detailed description with particular focus on the UHF-RFID Smart Gate implementation for the forklift action classification. In particular, with the term “action”, we refer to two particular movements that the forklift can do with respect to a UHF-RFID Gate. The *IN* action represents the forklift entering a certain area by crossing the gate. The *OUT* action, instead, refers to a forklift leaving a certain area by crossing the gate. The UHF-RFID Smart Gate proposed here is based on an asymmetrical deployment of the reader antenna to allow for a correct forklift discrimination with no additional sensors. The proposed system does not require calibration procedures, and it can be implemented with commercial-off-the-shelf (COTS) hardware. The designed classification method also presents a low computational burden. The Smart Gate implementation is described together with the performance evaluation of an on-site demonstrator. The paper is organized as follows: in Section 2, a state-of-the-art analysis of RFID Gates for good crossing identification is reported; Section 3 describes the I-READ 4.0 architecture, the UHF-RFID Smart Gate and the proposed phase-based action classification method; Section 4 shows the performance of the UHF-RFID Smart Gate, and finally, Section 5 sets conclusions and discusses some future developments.

## 2. RFID Gates

A UHF-RFID gate is usually composed of a UHF-RFID reader connected to one or more antennas and possibly with other optional devices. Typically its main task is the identification of crossing tagged assets, being goods, people, or vehicles, such as forklifts or pallet trucks. However, an RFID gate able to provide the direction of transit of the identified object/person, can allow a complete awareness of the asset locations in plants or warehouses.

Typically, two main problems occur when deploying an RFID gate in an industrial environment. First, due to the large beamwidth of standard reader antennas and the multipath effects typical of an indoor scenario with metallic objects and surfaces, the target assets crossing the gate are identified together with other static or moving tagged items nearby the gate, so stray read events may occur [13]. Second, the tag reading rate can be slowed due to the presence of the other tags demanding the communication channel resources, thus introducing a non-null probability that the tag on the target asset does not respond to any interrogation query during the crossing action [14]. The multipath effect could also affect the correct detection of the target RFID tags due to the fading effect of the communication channel [15]. To mitigate these issues, solutions relying on shielded reading zones using tunnel gates [16] were proposed. However, such solutions are required for a strong modification of the work environment and are not always suitable or easy to deploy. To avoid shielding structures, other solutions were proposed in [17,18,19]. In [17], a localization technique is combined with the gate functionality to solve the problem of discriminating among moving and static tags. Keller et al. [18] suggested using various aggregated attributes based on the low-level reader data, e.g., Electronic Product Code (EPC), Received Signal Strength Indicator (RSSI), timestamp, and reading antenna, to perform a classification algorithm in forklift truck applications, getting an overall accuracy of 95.5%. To improve the performance, the same authors extended the method by using an advanced reader antenna setup [19]. By employing a portal configuration with two readers and eight antennas, an overall accuracy of around 99% is obtained at the expense of a relatively high infrastructure cost.

To determine the crossing direction of the assets, additional devices such as light or ultrasound motion sensors [20] can be used, despite the high complexity and cost of the system. Moreover, light or ultrasound motion sensors are prone to false-positives or interruptions as unexpected entities obstruct the sensors. Other systems may employ Computer Vision (CV) and RFID systems as the concept presented in [21], but CV may give rise to privacy issues and also suffers from the outage problem if the light conditions of the environment are not adequate.

To limit cost and complexity of the system, solutions based only on RFID technology have been proposed. The first systems employed more than one antenna to estimate the crossing direction of assets by processing the detection information and the RSSI measurements. In [22], a method was proposed that uses the difference in the crossing time of two antennas aligned along the gate crossing direction without additional external sensors. In [23], a similar method was proposed relying on active RFID tags and based on creating different interrogation zones for each antenna. In [24], a double antenna scheme to control the access of children at a school door was proposed. The antennas are placed on the school door, one facing the inside, the other the outside.

Phase-based solutions [25] can be useful as the backscattered signal phase varies significantly with the motion of tagged assets, and can be profitably used to allow the usage of a single antenna, thus reducing the infrastructure cost. An example of an RFID phase-based access control system exploiting a single antenna was presented in [26] for tagged people crossing-direction discrimination. It is noteworthy that phase-based techniques can also allow to discriminate tags carried out by a forklift [27] or moving along a conveyor belt [28] with respect to static tags in the warehouse/plant scenario. The concept of phase measurements applied to conveyor belts was also explored in [29], where a two-antenna architecture was proposed for measuring the Direction of Arrival (DoA) of moving RFID tags for localization purposes. The Doppler Effect can be indeed profitably exploited for the tag localization on conveyor belts, as demonstrated by [30].

More recently, machine learning techniques were investigated in RFID systems both for localization purposes [31,32] and RFID Smart Gate implementation [33,34,35]. In [33], a single antenna architecture was proposed to determine the direction of people crossing an indoor RFID gate based on an Artificial Neural Network (ANN). Consecutive RSSI data are aggregated within frames, and the mean RSSI for each time frame is fed as an input feature for the neural network. The obtained accuracy is higher than 99%. Machine Learning solutions were also employed to solve the issue of stray reads [34], where a 97.5% classification accuracy among actual RFID tags crossing the gate and static or other tags moving close to the gate without crossing it was achieved with a single antenna architecture. However, such a system does not allow the crossing direction estimation. In [35], both the RSSI and the phase are processed through different machine learning techniques to discriminate among moving and static RFID tags. In fact, when the relative distance between the reader antenna and the tag changes, both the received power and phase change significantly.

A concept for asset tracking was proposed in the patent [36] as a device-free user localization scheme. Basically, a set of antennas is attached at the ceiling facing the floor, whereas a set of tags is placed on the floor. A moving object can shadow the tags and create a signature of the motion of the object itself. The same scheme is applicable for RFID gates, as proposed in [37,38] to solve the problem of pallet trucks crossing a key point (e.g., to monitor the charging of goods on a truck). In both solutions, an antenna is placed at the ceiling facing downwards, and a regular grid of 24 tags is placed on the floor. When a metallic cart crosses the target area, the tags are shadowed. Such information is given in input to a Long-Short Term Memory (LSTM) [39] Recurrent Neural Network (RNN) [37] or a convolutional neural network [38]. In both cases, a classification accuracy of 100% is obtained. Despite the robustness of these solutions, the deployment of the tags on the floor is unfortunately not always possible in warehouse scenarios, as the tags cannot stand high pressures caused by the weight and encumbrance of industrial vehicles such as forklifts.

## 3. Materials and Methods

### 3.1. The I-READ4.0 System Architecture

**Figure 1:**
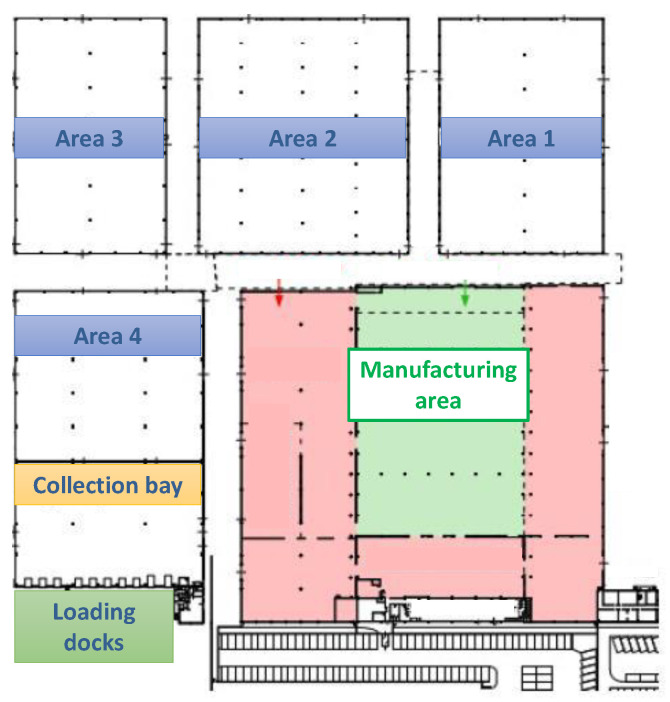
*Tassignano* warehouse plan.

**Figure 2:**
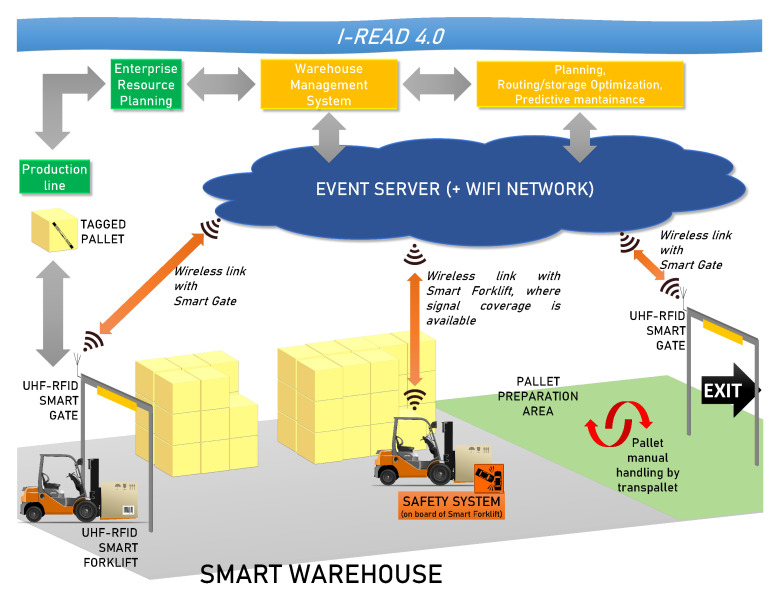
The I-READ 4.0 framework.

The I-READ 4.0 system was conceived by considering large-area warehouses with a high pallet-handling per day. The demonstrator was designed to operate in the *Tassignano* warehouse of the Sofidel paper industry with headquarters in Porcari, Lucca (https://www.sofidel.com/, accessed on 26 May 2021). It has an area of around 20,000 m^2^ (Figure 1) with an average handling of 2000–3000 pallets per day. Figure 2 illustrates the I-READ 4.0 framework, which comprises two main technological elements: the UHF-RFID Smart Gate (studied in this paper) and the UHF-RFID Smart Forklift.

The general architecture of the system is briefly described here. The proposed solution uses the passive UHF-RFID technology and particularly an integrated network of RFID readers, some fixed (UHF-RFID Smart Gates) and other mobile (UHF-RFID Smart Forklifts), capable of identifying individual pallets, their status (loaded by a forklift or unloaded), and their location (Figure 2). The tagged objects are pallets containing the final product, e.g., tissue paper. The pallets exit from the end of the production line and are brought into the storage warehouse carried by forklifts. Again, when the product must be shipped, a forklift lifts the pallet and brings it to the loading area (pallet preparation area). Then, each single pallet is loaded onto the truck manually handled by a pallet truck. For the correct management of the warehouse, it is essential to trace all these steps. Both the warehouse entrance at the end of the production line and the exit are equipped with a UHF-RFID Smart Gate, described later in this manuscript, which is capable of monitoring all the access/departure of products and forklifts to/from the warehouse. When the UHF-RFID Smart Forklift moves inside the warehouse, it is localized with a tracking system to allow the real-time pallet localization. In fact, the pallet location is associated to the forklift location at the time of the unloading event. In this context, the presence of the UHF-RFID Smart Gates can be fruitfully exploited to set the initial position of the forklift when developing tracking systems. Through the Wi-Fi network, the Smart Gate and the Smart Forklift send the data regarding position and status of each pallet to the warehouse central server. The knowledge of the position of pallets and forklifts allows to produce a real-time map of warehouse occupation and therefore enables to implement an optimization algorithm to improve the management of good flows and the occupation of warehouse areas. Furthermore, the information on the forklift position, combined with the data of the collision detection system installed on each forklift, allows to carry out a statistical analysis about the areas with the highest risk of collision. The detection of these potential collisions (near miss) will be shown to the forklift drivers and the Warehouse Management System (WMS) through the Event Server. For the aim of this paper, the design, development and testing of the UHF-RFID Smart Gate are relevant. That is, we are going to focus on that component of the global system architecture.

**Figure 3:**
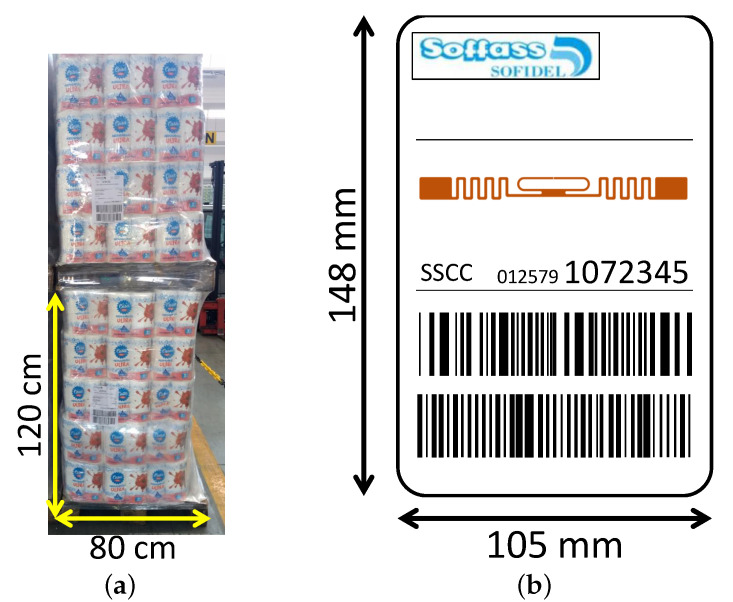
(**a**) Column composed by two tagged pallets and (**b**) sketch of the tagged label applied on the pallet (the tag is on the label rear side).

**Figure 4:**
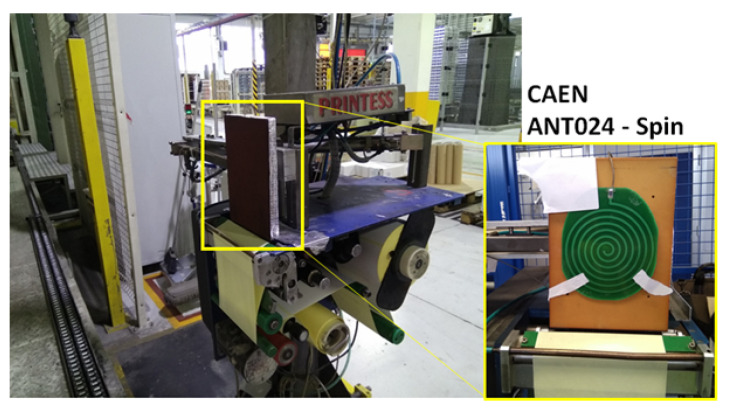
RFID label printer at the end of the production line.

Items coming out from the production lines are assembled in pallets. Each pallet is around 80 ×120 cm wide, and it has to be equipped with an identification label of size 148 ×105 mm according to the Global Standard GS1 (Figure 3). The label is printed at the end of the production line and shows the Serial Shipping Container Code (SSCC). Behind the label, there is a UH101 tag by LAB-ID measuring 95 ×88 mm and equipped with the NXP UCODE 7 chip (http://www.lab-id.com/wordpress/wp-content/uploads/2017/06/UH101.pdf, accessed on 26 May 2021) with −21 dBm sensitivity. The tag on the *smart label* is initialized through the CAEN RFID Proton R4320P reader (https://www.caenrfid.com/en/products/proton-r4320p/, accessed on 26 May 2021) connected to the CAEN RFID ANT-024 SPIN antenna. A picture of the end of the production line along with the RFID hardware to write the tag EPC is shown in Figure 4. In particular, the EPC is properly derived from the translation of the SSCC code according to the GS1 standard (https://www.gs1.org/sites/default/files/docs/epc/EPC-RTIPalletTagging-ImpGuide-i2.pdf, accessed on 26 May 2021).

Pallets are taken from the manufacturing area by Laser Guided Vehicles (LGVs) and carried at the entrance of the warehouse, which is composed of four storage areas (Figure 1). Here, workers handle them through the RFID Smart Forklift and bring them to a specific warehouse area passing through the RFID Smart Gate.

### 3.2. The UHF-RFID Smart Gate

Within the I-READ 4.0 system, the goal of the UHF-RFID Smart Gate is to monitor the crossing of goods at a point of interest within the warehouse. The gate must be able to completely identify the loaded pallets carried out by the forklift, to identify the forklift, and to understand its crossing direction. In fact, most gates can be crossed in both directions, and it is important to correctly determine if a product enters or leaves a certain warehouse area. That is, the UHF-RFID Smart Gate must implement an action classification method to understand whether the forklift is entering (IN) or leaving (OUT) a certain zone. The UHF-RFID Smart Gate is composed of the following hardware:

- An Impinj Speedway Revolution R420 UHF-RFID reader;
- A circularly-polarized (CP) CAEN WANTENNAX019 antenna;
- A linearly-polarized (LP) CAEN WANTENNAX007 antenna;
- Two coaxial cables;
- An ethernet cable to connect the reader to the Event Server;
- A power supply for the reader.

The circularly-polarized (CP) antenna CAEN WANTENNAX019 (https://www.caenrfid.com/it/products/wantennax019/, accessed on on 26 May 2021) was installed in the upper part of the gate, at a height of about 4.5 m. It has a gain of 8.5 dBc and a half power beam width of 65° on both planes ($HPBW_{H}=HPBW_{V}=65{}^{\circ}$). It was installed with a tilt angle of about 30° with respect to the horizontal plane to create an asymmetrical radiation-pattern footprint with respect to the gate. Thanks to this particular configuration characterized by an asymmetrical antenna deployment, the forklift crossing direction can be determined by using only a single antenna, as described later. Such CP antenna is mainly used to identify the forklift tags and to perform the action classification method.

**Figure 5:**
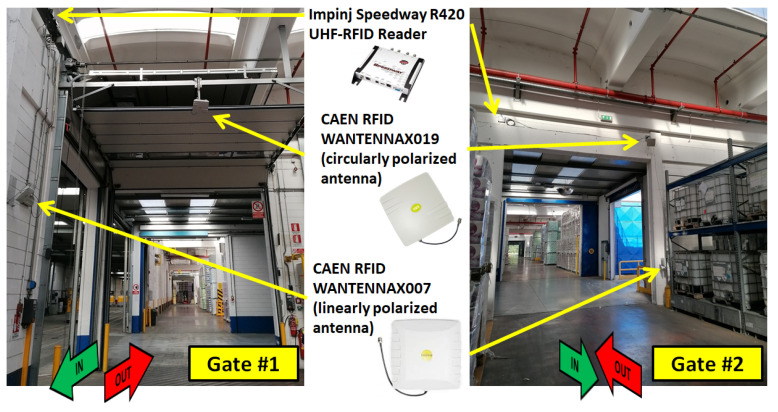
RFID Smart Gates installed at two entrances of the *Tassignano* warehouse.

With the intention of increasing the reliability of the gate when detecting all the carried pallets, a second antenna was installed at the gate side. Since the RFID labels on the pallets are always applied at the same position and parallel to the ground, a linearly-polarized (LP) antenna was chosen to maximize the power radiated to the tag. The chosen model is the CAEN WANTENNAX007 (https://pdf.directindustry.com/pdf/caen-rfid/wantennax007/113435-366469.html, accessed on on 26 May 2021) with gain equal to 8.0±0.5 dBi, and half power beam width equal to 65° on the horizontal plane ($HPBW_{H}=65{}^{\circ}$) and 68° on the vertical plane ($HPBW_{V}=68{}^{\circ}$). The antenna was fixed to the wall at a height of about 3 m from the ground and tilted to about 45° with respect to the horizontal plane. In Figure 5, two of the UHF-RFID Smart Gates installed at the warehouse entrance are shown. It must be highlighted that the gate infrastructure does not include additional invasive metallic structures as typical for tunnel gates [16].

**Figure 6:**
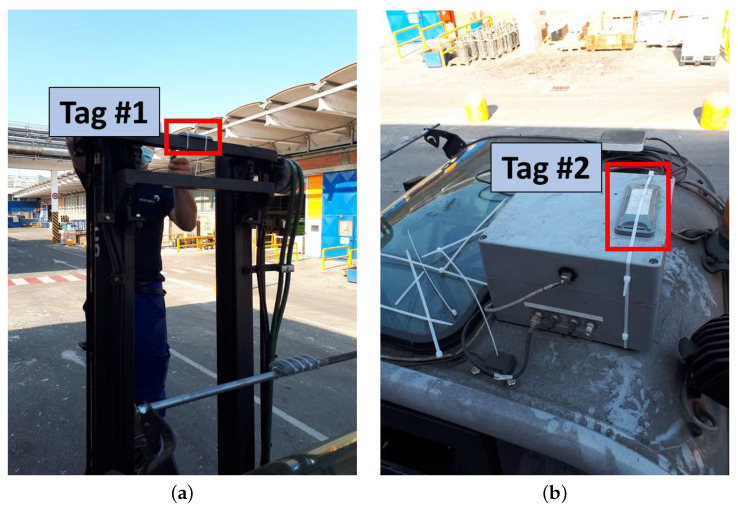
RFID tags placed on the forklift. (**a**) Tag placed on the upright, and (**b**) tag placed on the forklift top.

The forklifts are equipped with two OMNI-ID EXO 2000 on-metal RFID tags (https://omni-id.com/datasheet/1373, accessed on 26 May 2021) to be identified by the gate. One tag is placed on the forklift upright at a height of 2.6 m (Figure 6a), while the second tag is placed on the forklift roof at a height of 2.2 m (Figure 6b) for redundancy purposes.

**Figure 7:**
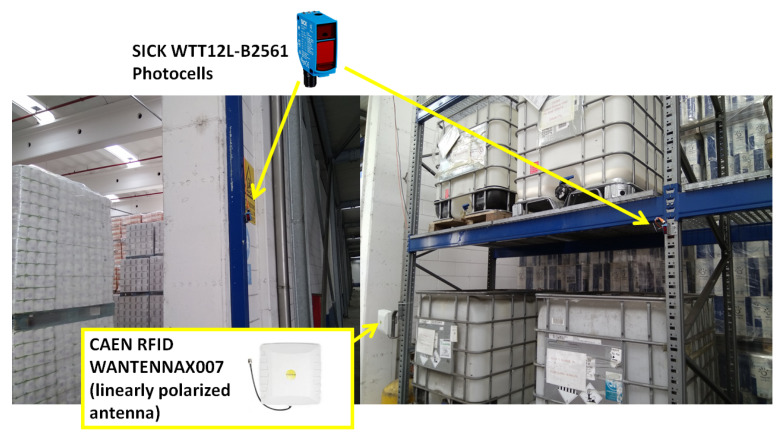
Photocells installed at one of the UHF-RFID Smart Gates.

Two photocell barrier sensors SICK WTT12L-B2561 are placed in proximity of the gates to evaluate the performance of the phase-based action classification method and to get an estimate of the forklift speed *v*. A picture of the photocells is in Figure 7.

### 3.3. Signal Model

The phase-based action classification method proposed here enables a smart-gate operation with a single antenna to determine the moving direction of the forklift crossing the gate. When the reader interrogates a tag, the latter backscatters the impinging signal, thus enabling the reader to measure a phase proportional to the distance between the reader and the tag. When the tagged forklift crosses the gate, the reader antenna performs several queries of the moving tag and measures the phase of the signal at different time steps $t_{n}$, being $n\in \{0,...,N_{R}-1\}$ and $N_{R}$ the number of successful queries of the tag during the vehicle crossing. To be more precise, the phase of the signal measured by the reader at time $t_{n}$ can be resumed as:

(1) $$\phi \left(t_{n}\right)=\mathrm{mod}{\left(\frac{-4\pi d(t_{n})}{\lambda }+\phi_{0}\left(\theta_{R},\psi_{R},\theta_{T},\psi_{T},t_{n}\right)+\phi_{m}\left(t_{n}\right)\right)}_{2\pi }$$

where $d(t_{n})$ is the distance between the tag and the reader at time $t_{n}$, λ is the carrier wavelength in free-space, $\phi_{0}\left(\theta_{R},\psi_{R},\theta_{T},\psi_{T},t_{n}\right)$ is the phase bias caused by reader and tag antennas and by the electrical circuitry, where $\theta_{R}$ and $\psi_{R}$ are the elevation and azimuth angle at time $t_{n}$, respectively, at the reader antenna side, and $\theta_{T}$ and $\psi_{T}$ are the elevation and azimuth angle at time $t_{n}$, respectively, at the tag antenna side. $\phi_{m}\left(t_{n}\right)$ is the contribution to the phase caused by multipath phenomena at time $t_{n}$. The distance $d(t_{n})$ is defined as:

(2) $$d\left(t_{n}\right)=∥p_{\mathrm{ant}}-p_{\mathrm{tag}}\left(t_{n}\right)∥\;$$

where $p_{\mathrm{ant}}$ is the vector ${\left[x_{ant},y_{ant},z_{ant}\right]}^{T}\in R^{3}$ of the reader antenna location, and $p_{\mathrm{tag}}\left(t_{n}\right)$ is the vector ${\left[x_{tag}\left(t_{n}\right),y_{tag}\left(t_{n}\right),z_{tag}\left(t_{n}\right)\right]}^{T}\in R^{3}$ of the tag trajectory sample at time $t_{n}$.

The value of $\phi_{0}\left(\theta_{R},\psi_{R},\theta_{T},\psi_{T},t_{n}\right)$ is defined as:

(3) $$\phi_{0}\left(\theta_{R},\psi_{R},\theta_{T},\psi_{T},t_{n}\right)=\phi_{TX}\left(\theta_{R},\psi_{R},t_{n}\right)+\phi_{RX}\left(\theta_{R},\psi_{R},t_{n}\right)+\phi_{tag}\left(\theta_{T},\psi_{T},t_{n}\right)$$

where $\phi_{TX}$ and $\phi_{RX}$ are the phase offsets caused by the transmitting and receiving circuitry of the reader, and $\phi_{tag}$ is a phase offset that depends on the tag itself and may be different even among tags of the same model. The $\phi_{0}\left(\theta_{R},\psi_{R},\theta_{T},\psi_{T},t_{n}\right)$ term is almost constant over consecutive tag query responses within the reader antenna’s main beam, and it will be indicated in the rest of the paper as $\phi_{0}$.

To overcome the problem of the phase 2π − ambiguity, we can perform phase unwrapping [40]:

(4) $$\phi^{u}\left(t_{n}\right)=\frac{-4\pi d(t_{n})}{\lambda }+\phi_{0}+\phi_{m}\left(t_{n}\right)$$

To correctly execute the phase unwrapping, consecutive phase samples must not differ more than π. If we consider the value of $\phi_{m}\left(t_{n}\right)-\phi_{m}\left(t_{n-1}\right)\approx 0$, meaning that the phase difference caused by the multipath between consecutive time steps is negligible, only the condition $d\left(t_{n}\right)-d\left(t_{n-1}\right)<\lambda /4$ must be satisfied. This fact is a direct consequence of the Nyquist–Shannon Sampling Theorem, which states the condition for which a signal is sampled without aliasing. Further considerations on the topic applied to the RFID field can be found in [41,42]. As it will be discussed later, a relatively high forklift speed or a poor RFID reader sampling rate may both lead to errors during the phase unwrapping process and, therefore, to classification errors.

Now, for the sake of simplicity, the value of $\phi^{u}\left(t_{n}\right)$ is normalized by the first sample acquired at n=0. We represent the normalized unwrapped phase with $\phi^{n}\left(t_{n}\right)$:

(5) $$\phi^{n}\left(t_{n}\right)=\frac{-4\pi \Delta d(t_{n})}{\lambda }+\Delta \phi_{m}\left(t_{n}\right)$$

where $\Delta d\left(t_{n}\right)=d\left(t_{n}\right)-d\left(t_{0}\right)$, and $\Delta \phi_{m}\left(t_{n}\right)=\phi_{m}\left(t_{n}\right)-\phi_{m}\left(t_{0}\right)$.

### 3.4. RFID Gate with Antenna in Symmetrical Configuration

**Figure 8:**
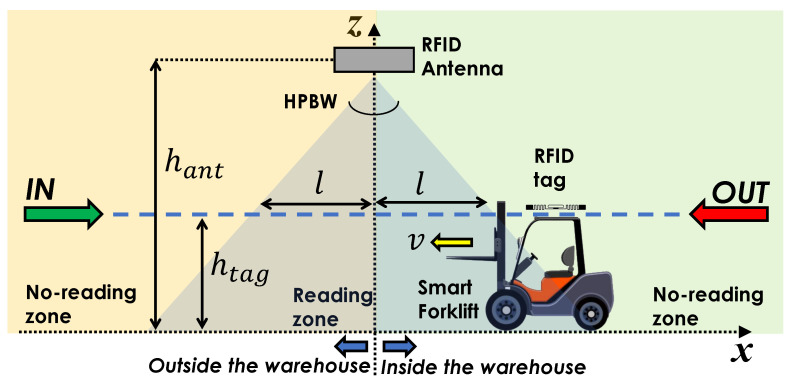
Sketch of the symmetrical configuration of the RFID Smart Gate.

By referring to Figure 8, we consider a bi-dimensional scenario in which the forklift moves mainly along the *x*-axis with a constant speed *v*; such a hypothesis is plausible in a few-second interval, when considering the forklift weight and inertia. When the forklift performs an *IN* action, it moves towards the positive direction of the *x*-axis with positive speed, whereas when performing an *OUT* action, it moves towards the negative direction with a negative speed. The tag is placed on the forklift top, at a height $h_{tag}$. The gate antenna is placed in ${\left[x_{ant},y_{ant},z_{ant}\right]}^{T}={\left[0,0,h_{ant}\right]}^{T}$, and it is facing the floor in such a way that its coverage area is symmetrical in the xy-plane with respect to the *z*-axis. The coverage area on the tag plane is determined by the antenna HPBW through the following equation:

(6) l=Δharctan(HPBW/2)

where Δh is the height difference between the antenna and the tag: $\Delta h=h_{ant}-h_{tag}$. This means that the tag is detectable when the forklift is inside the region |x|<l.

**Figure 9:**
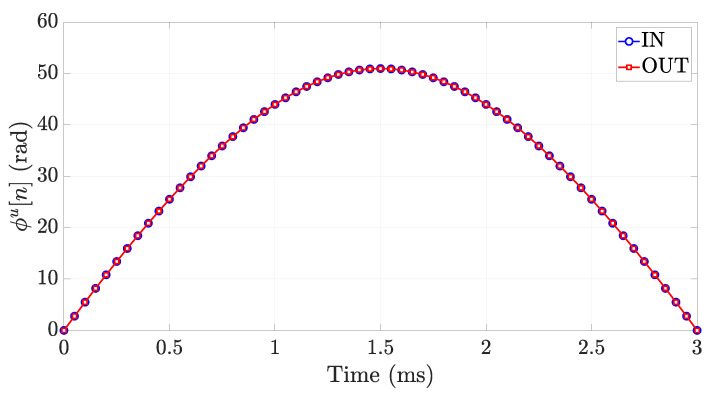
Time behavior of the unwrapped normalized phase in the symmetrical configuration of the RFID Smart Gate for the following system parameters: f=865.7 MHz, l=3 m, v=2 m/s, Δh=2.5 m, and T=50 ms.

Let us suppose the forklift is performing an *IN* action. The time variation of the *x*-coordinate is:

(7) x(t)=−l+vt

being t≥0. By considering a constant sampling time *T*, the acquisition time steps $t_{n}$ can be written as $t_{n}=nT$. By denoting $x\left[n\right]=x\left(t_{n}\right)=x\left(nT\right)$, we can also derive the normalized unwrapped phase sequence $\phi^{n}\left[n\right]$ with (4) as follows:

(8) $$\phi^{n}\left[n\right]=\frac{-4\pi }{\lambda }\left(\sqrt{{\left(-l+vnT\right)}^{2}+\Delta h^{2}}-\sqrt{{\left(-l\right)}^{2}+\Delta h^{2}}\right)$$

where we neglected the effect of the multipath for simplicity. Let us consider an RFID gate operating at the frequency f=865.7 MHz. The unwrapped normalized phase $\phi^{n}\left[n\right]$, is depicted in Figure 9 when l=3 m, v=2 m/s, Δh=2.5 m, and T=50 ms, for both *IN* and *OUT* actions. As expected, during an *IN* action, the normalized unwrapped phase decreases when the forklift (tag) is approaching the antenna in the region x≤0, while it increases once the forklift (tag) has crossed the gate and gets further from the antenna in the region x>0. For the *OUT* action, instead, the normalized unwrapped phase decreases when the forklift (tag) is approaching the antenna in the region x≥0, and increases once the forklift (tag) has crossed the gate and gets further from the antenna in the region x≤0. It appears straightforward that the time behavior of $\phi^{n}\left[n\right]$ is the same for both *IN* and *OUT* actions, as the antenna coverage area is symmetrical. Therefore, it is not possible to discriminate between the two actions by using this gate configuration.

### 3.5. RFID Gate with Asymmetrical Antenna Deployment and Action Classification Method

**Figure 10:**
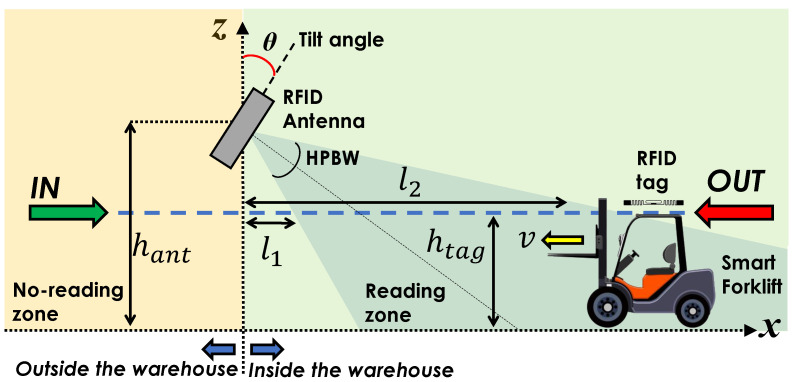
Sketch of the asymmetrical gate.

**Figure 11:**
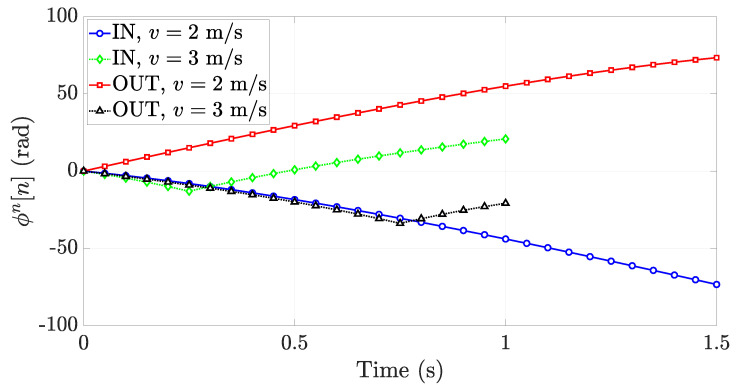
Time behavior of the normalized unwrapped phase in the asymmetrical antenna deployment for *IN* and *OUT* actions by varying the speed *v* when the parameters are the following: f=865.7 MHz, $l_{1}=1$ m, $l_{2}=4$ m, Δh=2.5 m, and T=50 ms.

To make the $\phi^{n}\left[n\right]$ time behavior different between the two actions, *IN* and *OUT*, and to allow correct action discrimination, the reader antenna is tilted of an angle θ with respect to the vertical axis (*z*-axis) to make it point towards the inside of the warehouse in such a way that the reader cannot detect tags outside the room, as shown in Figure 10. Let us suppose that the antenna is pointed in such a way that it can only detect tags within the region $l_{1}\leq x\leq l_{2}$, with $l_{1}$ and $l_{2}$ real positive values and $l_{1}<l_{2}$. When the forklift performs an *IN* action, the tag will be detected only when it is getting further from the antenna, so the $\phi^{n}\left[n\right]$ will be a decreasing function. On the other hand, when the forklift performs an *OUT* action, the tag will be detected only when it is getting closer to the antenna, so the $\phi^{n}\left[n\right]$ will be an increasing function. The time behavior of $\phi^{n}\left[n\right]$ for *IN* (blue circular markers) and *OUT* (red squared markers) actions is depicted in Figure 11 when $l_{1}=1$ m, $l_{2}=4$ m, v=2 m/s, Δh=2.5 m, and T=50 ms. These results confirm that the asymmetrical configuration of the gate antenna guarantees the capability of recognizing the *IN* and *OUT* actions, without requiring additional antennas or sensors.

The classification algorithm is straightforward. If the measured $\phi^{n}\left[n\right]$ is a decreasing function, the estimated action is *IN*; otherwise, the estimated action is *OUT*. To do that, we first interpolate the measured curve with a first-order polynomial function. Then, we calculate the slope coefficient *m* and execute the following decision criterion:

(9) $$\begin{array}{c} \left\{\begin{array}{cc} \mathrm{Classified}\;\mathrm{action}:\;\mathrm{IN} & \mathrm{if}\;m\leq 0,\\ \mathrm{Classified}\;\mathrm{action}:\;\mathrm{OUT} & \mathrm{if}\;m>0 \end{array}\right. \end{array}$$

As already said, to operate correctly, this algorithm must rely on a correct phase unwrapping of the measured phase. When the forklift speed increases, the average spatial sampling may be greater than λ/4. This effect makes the Nyquist sampling condition not satisfied, and the slope of the normalized unwrapped phase may change at some points. By leaving all the other parameters unchanged, Figure 11 also shows the normalized unwrapped phase for the forklift speed v=3 m/s, instead of v=2 m/s. The aforementioned slope change is strongly evident for both the *IN* (green diamond markers) and *OUT* (black triangle markers) actions. This means that, on the basis of the forklift speed, the estimation of the curve slope *m* could fail by leading to a possible classification error. As a consequence, the reader queries have to be sent with a time interval able to guarantee the Nyquist sampling condition by knowing the maximum allowed speed for the forklift.

As we will see in the next section, the influence of the environment can also introduce errors in the classification method.

Moreover, static tagged forklifts or pallet tags nearby the gates can be filtered out from the classification method, as their measured phase is almost constant. An advantage of this algorithm is the low-effort computational burden which allows the method implementation on low-power computers, as it will be shown in the next section. Alternatively, the method can be directly executed on an RFID reader dedicated PC if this is present. Another solution is to transmit the data on an external PC that controls all the RFID Smart Gates of the warehouse, as was done in this proof of concept.

## 4. Experimental Analysis

### 4.1. Experimental Results

**Figure 12:**
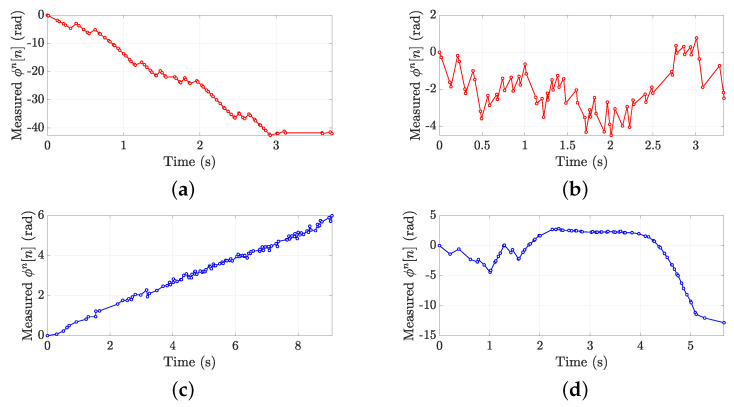
Examples of measured normalized unwrapped phase $\phi^{n}\left[n\right]$. (**a**) Successful *IN* classification, (**b**) wrong *IN* classification, (**c**) successful *OUT* classification, (**d**) wrong *OUT* classification.

Figure 12a,b shows an example of a successful and unsuccessful *IN* classification, respectively. As apparent in Figure 12b, the unwrapping fails by causing a wrong sign estimation of the slope coefficient *m*. Similarly, Figure 12c,d shows an example of a successful and unsuccessful *OUT* classification. In such a case, the slope coefficient *m* of Figure 12d is wrongly estimated as negative. Table 1 resumes the principal features of the showed curves in terms of the number of samples $N_{R}$, time duration of the crossing action $T_{d}$, forklift speed *v*, average sampling time $\Delta_{T}$, average spatial sampling $\Delta_{S}$ and measured slope *m*.

There are multiple causes of unsuccessful classification, mainly related to a failed phase unwrapping in industrial scenarios. First of all, the multipath phenomena can introduce strong and unpredictable contributions on the phase variation $\phi^{n}\left[n\right]$. Second, the presence of multiple tags close to the gate demanding for the communication channel may slow the forklift-tag reading rate. Finally, the speed of the forklifts, which can move up to 3.5 m/s, may cause a poor sampling of the phase curve and consequently a wrong phase unwrapping.

To better understand the effect of the forklift speed *v* and evaluate the classification accuracy, an experimental campaign was conducted. We analyzed a total of $N_{T}=264$ trials acquired from the gate placed at the production line end during the regular forklift operations. The number of recorded *IN* and *OUT* actions is $N_{IN}=164$ and $N_{OUT}=100$, respectively. The reason for such difference is due to the exclusion from the experimental analysis of all the cases where the optical barrier sensors failed, so it was not possible to determine the forklift speed and recognize the ground truth of the forklift passage. In 100% of the cases, at least one of the two tags placed on the forklift was detected by the CP antenna at least once.

**Figure 13:**
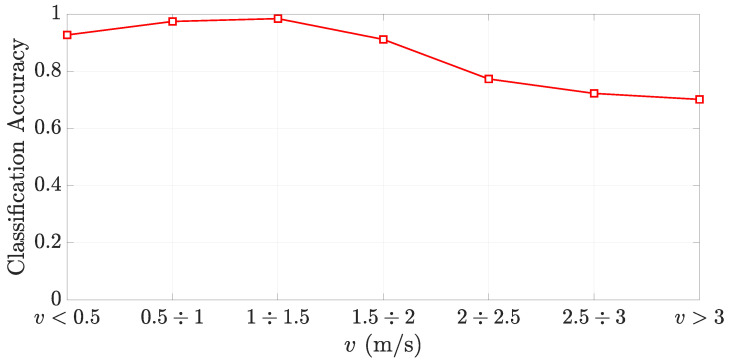
Classification accuracy vs. forklift speed *v*.

The classification accuracy computed for different ranges of the forklift speed *v* is shown in Figure 13. The overall action classification accuracy of the method is 92% but reaches a maximum value of 97–98% when the forklift travels at a speed between 0.5 m/s. and 1.5 m/s. It is apparent that, when the forklift overpasses the speed of 1.5 m/s, the accuracy of the action classification method decreases as the phase unwrapping fails. On the other hand, a low forklift speed can be detrimental too, since the phase slope could be too close to zero, making the action classification less reliable. This effect is apparent in Figure 13 for v<0.5 m/s.

**Figure 14:**
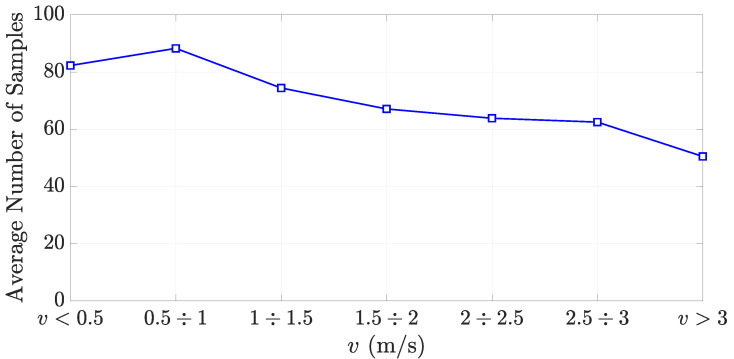
Average number of samples vs. forklift speed *v*.

The number of tag readings is a crucial parameter for the success of the classification algorithm. Therefore, the average number of samples with respect to the forklift speed is also reported (Figure 14). As expected, the number of available readings decreases with the increase of the forklift speed. However, thanks to the proper reader configuration, the average number of readings never goes under 45 for v<3 m/s. When this cannot be guaranteed, ad-hoc interpolation techniques could be adopted.

**Figure 15:**
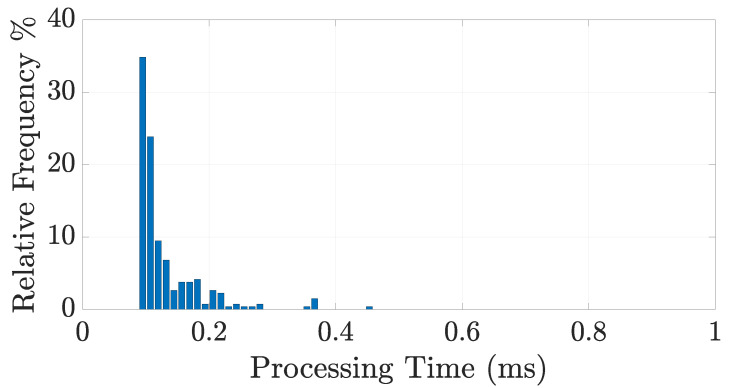
Histogram of the processing time (ms) for the $N_{T}=264$ analyzed trials.

Finally, to demonstrate the low computational burden of the proposed method, the elaboration time of the $N_{T}=264$ trials has been depicted in Figure 15. The analysis was conducted on a laptop with an Intel(R) Core(TM) i7-7700HQ CPU @ 2.80 GHz and 16 GB RAM, showing a mean elaboration time of 0.13 ms with a standard deviation of 0.05 ms. The case totality required less than 1 ms to be processed. Such a time is negligible with respect to the acquisition time, which depends on the forklift speed and can be in the order of 1–2 s. Therefore, we can conclude that the computational burden of the algorithm is not an issue at all.

### 4.2. Discussion

A discussion on possible alternatives to this algorithm must be conducted. As reported in [25], it is possible to measure the radial speed $v_{r}$ of a tag with respect to the reader antenna through the acquisition of the Doppler frequency shift. Indeed, the tag radial speed measurements in the asymmetrical antenna deployment can be profitably used for the forklift action classification similarly to (9). To obtain reliable Doppler frequency shift data, the reader manufacturer suggests to configure the Impinj Speedway R420 reader to low reading-rate modes [43]. In such way, the duration of the RFID signal packets is longer; therefore, the Doppler frequency shift is easier to be measured. However, such a condition does not fit with our need to have fast readings to ensure both the forklift and the goods detection and to satisfy the Nyquist–Shannon Sampling Theorem. Therefore, during the tests, we had to configure the reader to a fast reading-rate mode, so the Doppler frequency shift measurements were affected by severe detrimental noise. Consequently, the here proposed signal processing Equation (9) resulted in a more robust, reliable and accurate action classification method. Additionally, the fast-rate reader configuration allows minimizing the number of cases where the Sampling Theorem is not met and phase unwrapping fails. Another aspect that must be considered is the Doppler shift $\Delta f=2fv_{r}/c$, when the forklift travels at high speed, e.g., v=3 m/s, Δf<17.31 Hz. Given that the bandwidth for a single RFID channel in the ETSI European lower band is 200 kHz [44], such Δf can be considered negligible and difficult to measure. Finally, the proposed method does not require any preliminary system calibrations, and can be implemented with COTS devices.

### 4.3. Comparison with the State-of-the-Art

Each state-of-the-art solution presented in Section 2 requires a different and custom architecture, so it is difficult to make a fair comparison by evaluating the classification performance of other pre-existing solutions directly on-site with the same antenna configuration and dataset. In any case, we can compare the proposed system with the others analyzed in Section 2 in terms of cost, encumbrance, and scalability. The cost of a COTS RFID system at the UHF-RFID band is mainly determined by the RFID reader, which may reach more than 1000$ (USD). Each RFID antenna costs around 100–200$ (USD) and, therefore, can be a significant cost for solutions requiring multiple antennas. The cost of a passive RFID tag can be considered negligible for small volumes of goods, as RFID inlay labels usually cost less than 0.1$ (USD). Some passive RFID tags designed for metallic surfaces can cost around 10–20$ (USD) each, but there are many models that can be bought for less than 5$. Battery-Assisted Passive (BAP), active, or sensor-equipped tags can reach a cost of 30$, but they are usually not necessary. Metallic supports or shields shall be included in the total cost of the system, and therefore, it turns out that shielded gates [16,18] are quite expensive solutions due to the large infrastructure required. The encumbrance is relative to the global volume occupied by the hardware needed to implement the gate, which could be very significant in the case of shielded gates. Cost and encumbrance together usually impact the scalability of the solution since a high cost, or alternatively, a high encumbrance, makes the solution less replicable inside the plant, factory, or warehouse. The scalability of a solution is also determined by the time required for the installation process. For instance, mounting several antennas at the ceiling, mounting several shielded gates, or installing several photocells or ultrasound barriers in addition to the RFID hardware could be a time-consuming operation, which must be considered as a significant cost. Finally, solutions based on a machine learning classification algorithm could require a supervised training process, which can be difficult to achieve in a short time, and huge amounts of data have to be collected in several operating conditions.

As summarized in Table 2, the solutions based on shielded gates [16,18] have been considered as “High Cost”, “High Encumbrance” and “Low Scalability” due to the cost of the metal shields, their volume, and the installation complexity. On the other hand, shielded gates are the best options to filter out false positive readings.

By referring to [19], we opted for “Medium–High Cost”, “Medium–High Encumbrance”, and “Low–Medium scalability”. Indeed, the proposed solution requires antennas aggregated in panels. The cost and the encumbrance are lower than the shielded gates, but the cost of the antenna panel is not negligible and must be considered when taking into account the system scalability. The systems proposed in [22,23,24] have been evaluated as “Low–Medium Cost”, “Low–Medium Encumbrance” and “Medium–High Scalability”. Indeed, the three systems require two antennas, which increase the cost with respect to solutions with a single antenna, and the encumbrance cannot be considered as “low”, too, as it is required to find enough space for two antennas. On the other hand, the installation of two antennas is indeed a fast process, and therefore, the scalability of the solutions is good. The solutions in [26,28] are based on phase processing, such as the one presented in this paper, and also require a single antenna. Therefore, they are classified as “Low Cost”, “Low Encumbrance”, and “High Scalability” [33] as they still rely on a single antenna, but the scalability is considered “Medium” as the proposed solution is based on a neural network classifier, which requires a time-consuming training stage. Following the same reasoning, the two solutions exposed in [37,38], both based on neural networks classifiers, are considered “Medium Scalability” solutions. In this case, however, the presence of the reference RFID tags on the ground makes the encumbrance of the solution higher with respect to solutions that do not require reference tags. Finally, the solution proposed in this paper is considered “Low Cost”, “Low Encumbrance” and “High Scalability”, as it needs a single antenna and does not require any calibration stages at the installation time. In comparison with the solutions of the same category in terms of cost, encumbrance, and scalability, e.g., [28], the proposed solution is designed to work in more complex environments with respect to the conveyor belt, where the speed of the RFID tags is known in advance, and the tag motion is constrained along assigned paths. Reference [26] is indeed a solution with low cost, low encumbrance and high scalability, but the proposed method has been evaluated only in a laboratory/office environment, whereas the solution proposed in this paper has been verified in a real industrial environment during regular work activities.

## 5. Conclusions

This paper presented an effective implementation of a UHF-RFID Smart Gate, a fixed identification point placed at warehouse key points for forklift monitoring. Each Smart Gate implements an action classification method that exploits the phase of the backscattering RFID signal to determine the gate crossing direction of the forklifts with respect to the gate. Thanks to an asymmetrical deployment of the reader antenna and the phase acquisition of the signal exchanged by the fixed reader antenna and tags on the forklifts, a scalable and low-cost solution exploiting only one antenna can be used for each gate, with no additional sensors. Performance and method capabilities were investigated through an experimental demonstrator installed in a real warehouse. Data were gathered during the regular operations of the workers. In 100% of cases, the forklift was detected by the RFID gate, and a 98% classification accuracy was achieved when the forklift speed ranged between 0.5 m/s and 1.5 m/s. The accuracy decreases for higher speeds. The proposed method requires short computational time and is therefore suitable for the real-time monitoring of the forklift crossings. For future developments, artificial intelligence techniques will be designed and evaluated to improve classification accuracy even when forklifts are moving at higher speeds.

## Figures and Tables

**Table 1:** Main features of the trials represented in Figure 12.

Trial	$N_{R}$	$T_{d}$ (s)	*v* (m/s)	$\Delta_{T}$ (ms)	$\Delta_{S}$ (cm)	*m* (rad/s)
Successful *IN*	85	3.7	1.57	44	7	−13.28
Wrong *IN*	72	3.3	0.98	46	4.5	0.1063
Successful *OUT*	114	9.1	0.49	80	3.3	0.62
Wrong *OUT*	76	5.7	0.77	74	5.7	−0.97

**Table 2:** Comparison of the proposed solution with the state-of-the-art.

Reference	Cost	Encumbrance	Scalability	Architecture
[16]	High	High	Low	Shielded Gate
[18]	High	High	Low	Shielded Gate
[19]	Medium–High	Medium–High	Low–Medium	Antenna Panels
[22]	Low–Medium	Low–Medium	Medium–High	One reader and two antennas
[23]	Low–Medium	Low–Medium	Medium–High	One reader and two antennas
[24]	Low–Medium	Low–Medium	Medium–High	One reader and two antennas
[26]	Low	Low	High	One reader and one antenna
[28]	Low	Low	High	One reader and one antenna
[33]	Low	Low	Medium	One reader and one antenna
[37]	Low	Low–Medium	Medium	One reader, one antenna, reference tags
[38]	Low	Low–Medium	Medium	One reader, one antenna, reference tags
This paper	Low	Low	High	One reader and one antenna

## Abbreviations

BC	Block Chain
COTS	commercial-off-the-shelf
CP	Circular Polarization
CPS	Cyber-Physical System
CV	Computer Vision
EPC	Electronic Product Code
HPBW	Half-Power Beamwidth
IoT	Internet of Things
LGV	Laser-Guided Vehicle
LP	Linear Polarization
LSTM	Long Short-Term Memory
NFC	Near-Field Communication
NN	neural networks
RFID	Radio Frequency IDentification
RNN	Recurrent Neural Network
RSSI	Received Signal Strength Indicator
SSCC	Serial Shipping Container Code
UHF	Ultra-High Frequency
WMS	Warehouse Management System
WSN	Wireless Sensor Networks
